# Techniques for the Detection of Sickle Cell Disease: A Review

**DOI:** 10.3390/mi12050519

**Published:** 2021-05-05

**Authors:** Wjdan A. Arishi, Hani A. Alhadrami, Mohammed Zourob

**Affiliations:** 1 Department of Medical Laboratory Technology, Faculty of Applied Medical Sciences, King Abdulaziz University, P.O. Box 80402, Jeddah 21589, Saudi Arabia; warishi0001@stu.kau.edu.sa; 2 Molecular Diagnostic laboratory, King Abdulaziz University Hospital, King Abdulaziz University, P.O. BOX 80402, Jeddah 21589, Saudi Arabia; 3 Department of Chemistry, Alfaisal University, Al Zahrawi Street, Al Maather, AlTakhassusi Rd, Riyadh 11533, Saudi Arabia

**Keywords:** sickle cell anemia, hemoglobinopathies, detection, diagnosis, point of care

## Abstract

Sickle cell disease (SCD) is a widespread disease caused by a mutation in the beta-globin gene that leads to the production of abnormal hemoglobin called hemoglobin S. The inheritance of the mutation could be homozygous or heterozygous combined with another hemoglobin mutation. SCD can be characterized by the presence of dense, sickled cells that causes hemolysis of blood cells, anemia, painful episodes, organ damage, and in some cases death. Early detection of SCD can help to reduce the mortality and manage the disease effectively. Therefore, different techniques have been developed to detect the sickle cell disease and the carrier states with high sensitivity and specificity. These techniques can be screening tests such as complete blood count, peripheral blood smears, and sickling test; confirmatory tests such as hemoglobin separation techniques; and genetic tests, which are more expensive and need to be done in centralized labs by highly skilled personnel. However, advanced portable point of care techniques have been developed to provide a low-cost, simple, and user-friendly device for detecting SCD, for instance coupling solubility tests with portable devices, using smartphone microscopic classifications, image processing techniques, rapid immunoassays, and sensor-based platforms. This review provides an overview of the current and emerging techniques for sickle cell disease detection and highlights the different potential methods that could be applied to help the early diagnosis of SCD.

## 1. Introduction

Sickle-cell disease (SCD) is a multisystem disorder related to acute illness, painful episodes, and gradual organ damage [1]. Sickle cell anemia is caused by point mutations in the HBB gene, which codes for β-subunit, where adenine is substituted by thymine (GAG > GTG) at codon 6 of the HBB gene. As a result of nucleotide substitution, the amino acid is altered, and glutamic acid is replaced by valine resulting in hemoglobin S (HbS) formation. Hb S polymerizes in a deoxygenated state and forms rigid, less soluble sickle-shaped cells [1,2]. SCD arises when inheriting two mutated alleles βS/βS (homozygous) or in the case of inheriting different types of mixed heterozygous alleles such as sickle-β-thalassemia HbSβ-thalassemia, sickle-hemoglobin C disease (HbSC), and other combinations. When the sickle cell trait (SCT)is heterozygous βA/βS, it means only one allele is affected and produces insoluble hemoglobin, and the other gene is wildtype and produces normal hemoglobin [3]. The pathogenesis mechanism of SCD depends on the polymerization of hemoglobin S, which is triggered by the lower oxygen affinity [4]. Polymerization alters the physical properties of red blood cells, such as shape and cell membrane, leading to dehydration of cells and increased polymerization. Repeated polymerization and sickling of cells lead to the formation of irreversibly sickle cell [4]. This accelerates cell destruction and reduces cells’ lifespan by ≥75%, resulting in hemolytic anemia [5]. In addition, the polymerized cell cannot move easily in the small blood vessels, resulting in the blockage of the vessel, i.e. vaso-occlusion [5,6]. The most common acute complication of SCD is acute vaso-occlusive crises (VOC) that cause pain crisis and acute chest syndrome, which is considered the major cause of hospitalization and death among SCD patients [4,7]. Chronic complications of SCD start to appear with age as organ failure due to the progressive ischemia leads to earlier death, cerebrovascular disease, pulmonary hypertension, retinopathy, and priapism. In addition, complications during pregnancy include preeclampsia and preterm delivery [8,9]. Children with SCD who live in Sub-Saharan Africa have a high mortality rate estimated at 50–80% by five years old. The most common cause of death in children is infection, including invasive pneumococcal disease and malaria [1,9]. In developed countries, the life expectancy of SCD patients has been improved by early diagnosis, comprehensive treatment, and general medical care. Therefore, early detection supports the effective management of the disease [10].

Detection of hemoglobin S and diagnosis of sickle cell disease depend mainly on the clinical laboratory, where a combination of biochemical and molecular tests is used in the detection and confirmation of the diagnosis [11]. The most popular methods for detecting these diseases are the full count of blood cells, Hb electrophoresis, and high-performance liquid chromatography (HPLC). These methods are considered the gold standard in the diagnosis of SCD [12].

## 2. Clinical Picture of the Inherited Hemoglobin Disorders

Sickle cell disease causes a variety of different illnesses; the most common disorder is sickle cell anemia HbSS with the genotype βs/βs. Other forms of the SCD are formed with a combination of βS mutation with other HBB mutations, such as sickle-hemoglobin C disease (HbSC) and sickle-β-thalassemia (either HbSβ+ or HbSβ0). β0 means there is no β-globin synthesis, while β+ means reduced production of β-globin [13]. The most severe forms of SCD are HbSS and HbSβ0, and they show same clinical picture. HbSC and HbSβ+ are considered the less severe forms of SCD [14]. The clinical picture of the sickle-β-thalassemia ranges from asymptomatic to severe state similar to HbSS sickle cell anemia [15], while, in some HbSC cases, severe and life-threatening complications will appear [16]. Some genetic factors can modify the sickle cell’s clinical expression when co-inherited with the βS gene, such as α-globin gene mutations, either one-gene deletion or two-gene deletion [17].

## 3. Techniques and Assays to Diagnose and Monitor SCD

Several techniques and assays are used for the detection and monitoring of the sickle disease. These techniques can be divided into two main categories: (1) currently used methods in the diagnosis of SCD; and (2) innovative techniques which are mostly still in the research stage. Several reviews have been published related to the development of point of care (POC) SCD detection [18,19,20]. Table 1 lists the different technologies developed for the diagnosis and monitoring of the SCD.

## 4. Current Techniques to Diagnose and Monitor SCD

### 4.1. Complete Blood Cell Count

The complete blood count (CBC) is a primary test to characterize the different types of anemia. However, the hemoglobin mutation will affect the hematological parameters, showing a variable change [20]. Patients with homozygous SS and heterozygous S/β° mutations usually present with hemolytic anemia where the red blood cells (RBCs), hemoglobin and hematocrit are low. In contrast, the counts of white blood cells (WBC) and platelet are elevated, and they can fluctuate. However, reticulocyte counts are variable and depend on different factors such as the degree of anemia caused by the cells hemolysis, sequestration, and bone marrow response to anemia [18,20]. Mean corpuscular volume (MCV) is usually elevated in SCD patients receiving hydroxyurea. Moreover, elevated red cell distribution width (RDW) is seen in SCD patients because of RBCs’ different subpopulations. Although CBC is widely used to describe the hematological parameter as valuable information, it is insufficient to give a complete picture of patients’ diagnoses [18,20]. 

### 4.2. Peripheral Blood Smear

The peripheral blood smear (PBF) is usually done after spotting abnormality in the automation counts and is considered a landmark of any hematological evaluation. PBF examines the morphology of the blood cell and evaluates any microscopic changes, which can provide valuable information that helps in the diagnoses of the different types of anemia [21]. In sickle cell anemia, moderate to severe anisopoikilocyte is seen with a variable number of elongated sickle cells, which is best observed when the red blood cells are deprived of oxygen [22]. The preparation of these blood smear slides is relatively simple, rapid, and inexpensive. Although peripheral blood smear is an informative hematological test, it relies on the pathologist’s skills, and the availability of trained pathologists is limited. Furthermore, the blood film analysis is too complicated due to the changes in the cell’s edge, location, shape, and size. As a result, a computerized system has been developed to provide a more accessible way to recognize the type of anemia [23].

### 4.3. Solubility Sickling Test

Sickling tests are mainly based on the polymerization of HbS in the deoxygenated state. The solubility test is the most widely used nowadays; its principle is based on the insolubility of Hb-S in the presence of concentrated phosphate buffer, a hemolyzing agent, and sodium dithionate. These agents crystalize the HbS and precipitate the cells, which refract the light and cause solution turbidity. The result is compared with negative and positive controls [24]. 

This test is easy to perform and inexpensive. It suffers from a false-negative result when utilized for newborns, due to the presence of a high amount of hemoglobin F and when the HbS is less than 10% of the total hemoglobin [25]. Furthermore, false-negative results are observed in patients with coinheritance of α-thalassemia trait and severe anemia. In contrast, false positives are observed in patients with high serum viscosity, erythrocytosis, highly marked leukocytosis and in some cases of anemia. Moreover, the sickle solubility tests cannot differentiate between sickle cells trait (SCT) and SCD, and they are insensitive to the detection of hemoglobin AS (HbAS)[25,26].These disadvantages make them difficult to use in screening programs [27].

### 4.4. Hemoglobin Electrophoresis

Electrophoresis is a type of chromatography techniques, and it is considered as one of the important tests used to detect Hb variants [11]. In this test, an electrical field is applied to facilitate the migration of electrically charged molecules. The first described hemoglobin variant Hb-S by using electrophoresis was in 1949. To identify hemoglobin variants, different pH and mediums are used, either cellulose acetate electrophoreses at alkaline pH or citrate agar at acidic pH [28]. 

Alkaline electrophoresis is a diagnostic tool that has been used to detect thalassemia and sickle cell anemia at pH 8.4. First, a hemolysate is prepared from the red blood cells; then, it is added to a cellulose strip and run-in buffer at a constant voltage in an electrophoresis chamber [29]. As a result, the different hemoglobin types with different net charges are separated into various bands depending on their mobility. Hemoglobin electrophoresis can differentiate between HbS and HbC, which are the most clinically significant variants. However, electrophoresis does not distinguish between hemoglobin variants with the same electrical charges and gives the same migration patterns, such as HbD and HbG, which comigrate with HbS; HbE and Hb0-Arab have similar migration to the HbC molecules [30]. Furthermore, alkaline electrophoresis can be affected by the presence of large amounts of hemoglobin F in newborns, which can dominate the smaller electrophoresis band. Therefore, extra care should be taken to reliably detect the HbS. In addition, smaller bands such as HbA2, HbH, and Hb Bart’s may be missed. Therefore, a more efficient test should be used as a diagnostic test to overcome these limitations [31].

Citrate agar electrophoresis is performed in acidic pH 6.0–6.2, and it depends on the interaction of the agaropectin in the gel mixture with the structural changes of the Hb [28]. Most hemoglobin variants that comigrate at alkaline pH can be separated effectively using citrate agar electrophoresis [32,33]. Citrate agar electrophoresis is not affected by the high amount of hemoglobin F in newborns; thus, it can be used as a diagnostic test for sickle cell disease at birth. However, it is laborious and challenging to perform in limited resources areas [28,34]. 

Capillary electrophoresis has been documented to separate Hb fractions and diagnose sickle cell disease and thalassemia. The capillary electrophoresis separates the protein in an untreated fused-silica column reliably [35]. Fully automated methods such as CAPILLARYS 2 system has been available in the market since the early 2000s. This method has eight parallel fused silica columns where multiple samples can be analyzed, and each column can be used for at least 3000 runs. The hemolysates are prepared automatically from red cell pellets [11]. The reference ranges for HbA2 are adapted to be 2.1–3.2% and <0.8% for HbF. However, in the presence of different Hb variants, Capillary Zone Electrophoresis (CZE) is better than HPLC for quantifying HbA2 except in the presence of HbC [36]. Moreover, a fully automated Neonat Fast Hb device with CAPILLARYS cord blood mode can analyze dried blood spots on filter paper and liquid cord blood. Thus, it can be used in the neonate screening test. These advantages make the CAPILLARYS instrument the first-line test for screening hemoglobinopathies in newborn and adult patients [36].

### 4.5. Isoelectric Focusing

Isoelectric focusing (IEF) is a high-resolution method for separating proteins depends on their isoelectric points (pI). The Hb molecules travel across a pH gradient until they reach their isoelectric points where the net charge is zero. The Hb molecules precipitate and appear as a sharp band [37]. This technique can detect HbS and HbA easily in a high concentration of HbF. Moreover, it separates Hb D-Punjab from HbS. Generally, it can provide the result within 45 min [37]. Although IEF is relatively expensive and requires highly trained personnel to interpret the results due to the larger number of bands, it is still considered the standard test for newborn screening, as it needs a very small volume of sample and can be used with a dried blood spot [38,39].

### 4.6. High Performance Liquid Chromatography

HPLC is documented to separate the hemoglobin fractions as they have different interaction with the stationary phase [40]. HPLC detects different types of hemoglobin based on the retention time and shape of the peak [41]. Each hemoglobin has a specific retention time and can be compared with the retention time of the known hemoglobin fractions [11]. HPLC is used to detect and quantify HbF, Hb A2, HbS, HbC, Hb Barts, and other Hb variants [11]. Developing a fully automated HPLC would be useful in testing a large number of samples accurately. HPLC shows better sensitivity in separation of hemoglobin variants than electrophoresis [42].

HPLC is much less labor-intensive and more reliable for monitoring patients under blood transfusion or hydroxyurea [43]. However, HPLC is an expensive machine and cannot differentiate among all variants with the same retention time. For example, all Hb variants with a similar retention time to HbS are eluted out with the HbS peak. Therefore, it can misdiagnose new variants that mimic HbS. Thus, HPLC cannot stand alone as a diagnostic test and should be done along with a confirmatory test such as DNA analysis before giving a final diagnosis [44].

### 4.7. Genetic Test

The genetic study is important for the precise detection of the various types of sickle cell disease, based on the detection of β-globin mutations that lead to sickle cell disease development [45].

#### 4.7.1. Polymerase Chain Reaction (PCR)-Based Techniques

Polymerase chain reaction is one of the most powerful diagnostic techniques, where special enzymes are used to amplify specific parts of the genetic materials to millions of copies, using specific primers. PCR can detect well known single genes or several genes in a single tube [46]. The PCR program involves denaturation, annealing, and elongation, which is repeated for 20–40 thermal cycles. Then, the result can be detected by gel electrophoresis, sequencing, melting curve analysis, or monitoring the change in the fluorescence. PCR sensitivity and specificity have revolutionized the prenatal and neonatal diagnostic field. Several PCR-based techniques are documented to detect βs mutations, such as high-resolution melting (HRM) analysis, which is simple, sensitive, and cost-effective for use in mass screening of SCD genotypes [47]. Another simple, low-cost PCR-based technique has been developed using bi-directional allele-specific amplification (ASA) and a hot star system to provide more specific single-tube genotyping, where the point mutation of sickle cell anemia is used as the SNP model. In addition, discriminatory conditions have enabled the determination of homozygous and heterozygous states based on the different band sizes on the agarose gel electrophoresis [46,48]. The amplification-refractory mutation system (ARMS) is a simple technique for detecting point mutation or small deletion. The ARMS principle is to use primers with specific sequences to allow the amplification of DNA in the presence of the target allele. Therefore, the detection of the target allele is based on the presence of the PCR product. The alleles can then be differentiated on agarose gel with different band sizes [49]. ARMS has been mostly used in prenatal diagnosis by detection of sickle cell mutation in the fetal sample. The ARMS’s sensitivity has been measured by comparing the result to identify the presence of hemoglobin variants by HPLC [50]. Wu et al. demonstrated an allele-specific oligonucleotide (ASO) hybridization to detect sickle cell mutation using two PCR primers. One primer was used for the normal allele and the other one for the mutated allele. The primer is joined to the complementary sequence and amplified, which in turn releases the fluorescent label that determines the amount of the target. This method can differentiate between the allelic variation [51].

#### 4.7.2. Restriction Fragment Length Polymorphism

**Figure 1:**
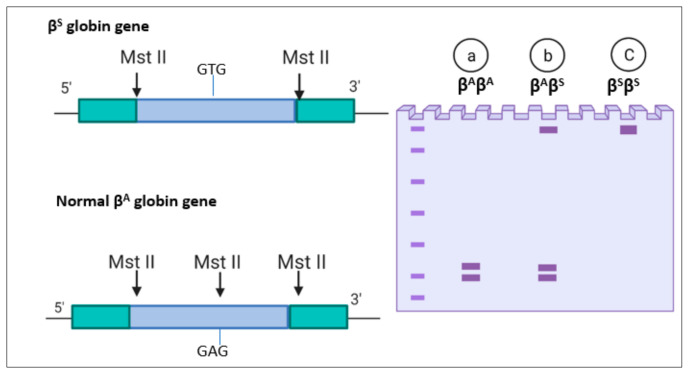
Restriction fragment length polymorphism (RFLP) for Sickle cell anemia: (**a**) normal gene(βAβA); (**b**) sickle cell trait (βAβS); and (**c**) sickle cell anemia (βSβS).

Restriction fragment length polymorphism (RFLP) is used to detect sickle cell disease based on restriction enzymes, which remove the recognition site at the βs mutated gene [52]. For example, MstII is one of the first described restriction enzymes; it cuts the DNA in the sequence CCTNAGG (where N represents any nucleotide). Therefore, when thymine replaces the adenine, it removes the recognition site for MstII restrictase, as shown in Figure 1. After separation, the number of bands resulting from the enzyme cutting indicates the number of mutations. In a healthy individual with (βA βA), the gene is cut by the MstII restrictase and yields two bands, as shown in Figure 1a. In homozygotes, the restrictase cuts both genes, and two short bands appear. In the sickle cell trait (βAβS), no cut is made in the βS, so a single band appears; however, the βA gene is cleaved, and two bands appear, as shown in Figure 1b. In sickle cell anemia homozygous (βSβS), there is no enzyme cutting due to the mutation in both genes, so a single wide band appears, as shown in Figure 1c [52]. Another restriction enzyme has been used in sickle cell detection is Ddel I. The mutation caused sickle cells Anemia(SCA) removes the restriction site of Ddel I, 5′-GTNAG-3′. As a result, bands with different lengths appear depending on the presence of sickle cell anemia mutation [53,54].

#### 4.7.3. DNA Microarrays and Sequencing Techniques

DNA microarrays consist of a large number of immobilized DNA oligonucleotide spots on the array surface, where hybridization events occur with complementary sequences, which in turn indicate the concentrations of the nucleic acids [55]. Microarrays have been used in genome-wide association studies (GWASs) to identify the presence of single nucleotide polymorphisms (SNPs) in a single run, as well as the copy number of variants [55,56]. Hamda et al. developed a novel database that combines the gene expression with genome-wide association study (GWAS), using homozygous SS microarray datasets to determine SCD transcriptomic profile [56,57]. 

Next-generation sequencing (NGS), which is deep DNA sequencing, has been used to identify different types of mutation. NGS can be run for the whole-exome sequencing (WES) or whole-genome sequencing (WGS). These techniques have been used widely for genetic analyses to predict sickle cell disease’s severity and progression, which can help make a treatment decision, discover new therapies, and develop novel diagnostic assays [58]. WES is performed to determine single-nucleotide variants (SNVs) in sickle cell mutation by sequencing the coding region of the β-globin gene. This procedure gives a full description of the β-globin gene accurately [59]. Few studies have utilized this approach to identify genetic modifications in the SCD severity. One study reported an increase in the number of strokes in African Americans due to mutation in GOLGB1 and ENPP1 [60]. Another study pointed out that mutation in SALL2 is associated with a high level of HbF in response to hydroxyurea [61]. Variants in MBL2 and KLRC3 were observed more frequently among adult SCD with hyperhemolysis syndrome than controls [62]. Whole-genome sequencing helps analyze the entire genome, identify the genomic modification of SCD, and create the Sickle Genome Project (SGP). It helped develop a robust pipeline for the correct identification of SNPs [63]. Furthermore, it confirmed the association of the SCD phenotypes with common genetic modifiers, including fetal hemoglobin BCL11A, HBB, UGT1A1, and APOL1. This technique will help in precision medicine to make better treatment decisions and discover new treatments [59].

## 5. Innovative Techniques for the Diagnosis and Monitoring of SCD

### 5.1. Image Processing Techniques

Image processing techniques play an essential role in the analysis of red blood cells. Blood cell disorders can be classified based on different features: the cell shape, central pallor diameter, target flag, etc. [64]. The cells can also be classified based on the image features by using segmentation and artificial neural network [65].

Chy and Rahaman developed an automated method to detect sickle cell anemia (SCA) using an image processing technique. An algorithm is used to automate the detection of sickle cells found in thin blood smears. The first step in this technique is to take blood images using a camera connected to a light microscope. Then, a pre-processing step converts the images into grayscale, enhances the image, and passes it through the median filter to reduce the noise. After that, the RBCs are segmented through a segmentation threshold, followed by a morphological operation for the image to remove the unwanted objects. The features of the images are created based on color, texture, and the cells’ geometry. As a final step, the computer classifier is trained to assess the picture. In total, 120 photos were used to assess this technique: 80 for training and 40 images for testing. The authors reported 95% accuracy and 96.55% sensitivity [66].

Alzubaidi et al. employed deep learning models to detect SCA and classify the red blood cells based on the microscopic images. The models were able to extract and implement the classification functions automatically in one shot. Moreover, they developed three deep learning models to determine and categorize the red blood cells based on the shapes: round shape indicating normal cells, elongated shape indicating sickle cells, and other blood shapes. The researcher focused on resolving the lack of training data, where they used the transfer learning technique. The study employed 626 images; 202 were classified as circular; 211 images were identified as elongated; and 213 as other cell shapes. The model achieved 99.54% accuracy and 99.98% when they used the same model plus a multi-class support vector machine [67]. 

De Haan et al. combined a smartphone microchip, a microscope, and machine learning algorithms to develop an affordable, portable, and rapid screening test for sickle cell anemia. This module uses two deep neural networks: The first one enhances the picture taken by the smartphone microscope. The second one complements the first neural network by enhancing the picture and performing semantic segmentation between the normal RBCs and sickle RBCs within the blood film. Finally, these segmented images are used to help the diagnoses of the sickle cell disease patients. This method achieved around 98% accuracy using 96 samples; 32 were SCD thin blood smears and 64 normal thin blood smears [22]. A smartphone-based image acquisition process has been developed for imaging the RBCs from the SCD patients under oxygen control. This method can automatically distinguish the normal RBCs from the sickled RBCs based on the morphology change, using image processing (MATLAB R2019a) to analyze the image and quantify the sickled cells. This advanced technique is cheap and easy to use [68].

The image processing methods provide automated interpreting of the blood cell images, minimizing errors, which can effectively monitor the SCD patient’s status [66,67]. However, the image processing techniques have some drawbacks: they cannot distinguish between the different types of the SCD; a high concentration of HbF can affect the polymerization of HbS, which can exclude the application of these tests to newborn screening [9]; they cannot classify RBCs accurately because they rely on binary classification, which ignores other blood cells shapes; and they are time consuming and require special equipment such as digital camera or smartphones [67].

### 5.2. Emerging Flow Cytometry

Conventional flow cytometry techniques have been used to detect sickle cells based on fluorescent markers or cellular morphology [69]. Advanced flow cytometry based on imaging techniques has been demonstrated to enhance the sensitivity by combining cell population analysis and morphological data. Beers et al. developed an imaging flow cytometry assay (SIFCA) and software algorithm to distinguish between sickle RBCs and normal RBCs based on their morphology. SIFCA is performed by diluting the peripheral blood sample, deoxygenating the cells by reducing the oxygen to 2% for 2 h, and then analyze it using imaging flow cytometry. Finally, the cells are classified based on the morphology into sickled and normal cells by using algorithm software. The authors analyzed 100 images of normal cells and 100 images of sickle cell, and they reported 100% sensitivity and 99.1% specificity. The study proved that SIFCA can assess sickling tendency in SCA patients to identify the severity of the disease and drug monitoring [70,71].

Cai et al. developed in-vitro photoacoustic flow cytometry (PAFC) for morphological detection of sickle cells containing hemoglobin S. They employed photothermal and photoacoustic spectra for determination of hemoglobin heterogeneity and accumulation of the HbS in sickle RBCs. The sickled RBCs showed 2–4-fold lower linear mode than normal RBCs. This method is useful in monitoring the sickling states [72]. Liua et al. developed microfluidic flow cytometry based on the electrical impedance spectroscopy. This technique detects the changes in the electrical impedance resulted from the change in the cells’ shape from the round soluble cells to sickle rigid cells under hypoxic condition. In this study, the cells were obtained from a healthy donor and three sickle cell patients, and the difference in the electrical impedance was measured to show the difference between normal cells and sickled cells. They showed that the electrical impedance signal can be used as an indicator of the cell sickling events. However, it is still unclear if these novel flow cytometry techniques can be used to monitor disease severity or if they can distinguish between sickle cell trait and sickle cell disease [19,73].

### 5.3. Mechanical Differentiation of Sickle Cells

The deformability of red blood cells is a crucial determinant of blood flow in circulation. In sickle cell disease, RBCs are mechanically fragile and poorly deformable, resulting in impaired blood flow. This feature of the sickle cell can be used to monitor the disease severity and the sickling event [74].

Brandao et al. developed an optical tweezer to capture red blood cells (RBC) by dragging them through a viscous fluid (human AB plasma) to measure the elasticity of the cells. In this study, the RBC deformability was measured in 10 homozygous patients (HbSS), 5 patients taking hydroxyurea (HU) for six months (HbSS/HU), 10 patients with sickle cell trait (HbAS), and 35 normal controls. The RBCs deformability was lower in the patients with HbSS and HbAS; however, in patients taking hydroxyurea HbSS/HU, the cells’ deformability was found to be similar to the normal control cells. These results show that optical tweezers have the potential to be used to monitor hydroxyurea response in sickle cell disease, but they cannot be used to distinguish between different types of hemoglobin diseases [74].

Qiang et al. demonstrated an amplitude-modulated electrodeformation in microfluidic for identifying the mechanical fatigue in single cells. This method depends on the cell’s mechanical fatigue, which leads to deterioration of the physical properties by subjecting the cells to static loads. In this method, a constant amplitude fatigue load is applied to deform the RBCs, and, with more fatigue cycles, the cells progressively lose their ability to stretch. Moreover, cyclic deformation was shown to be a fast method to deform RBCs under static deformation at the same maximum load. This testing platform can provide the possibility of flexible detection of sickle cells, but it is not yet validated in sickle cell detection [75].

Du et al. developed a microfluidic-based method to detect and quantify sickle cells by modulating the disease’s pathophysiology. In this study, the kinetics of cell sickling, unsickling, and cell rheology were investigated by exposing the cells to different hypoxic conditions to mimic microvasculature scenarios, sickling events, and hydroxyurea therapy. The microfluidic chip is a double-layer device consisting of a cell channel, polydimethylsiloxane film in the middle, and a gas channel where the O_2_ concentration is controlled. In the deoxygenated state, when the oxygen is less than 5%, the shape of the RBCs that contain HbS change, and form sickled cells within 12 s. This method has been used to monitor sickling events and hydroxyurea therapy [76].

Javidi et al. developed a spatiotemporal analysis of cell membrane fluctuations for the diagnosis of SCD. The test is based on using a hologram video containing either normal RBCs or sickled RBCs. The video was recorded using a low-cost, compact, 3D-printed shearing interferometer. Each hologram film was reconstructed and formed a spatiotemporal data cube. These data were extracted by calculating the standard deviations and the mean of the cell membrane fluctuations at every location over time. This resulted in a two-dimensional map of the standard deviation and mean. This method can be considered as low-cost, fast, and it does not need trained personnel to run. The accuracy of the results could be enhanced by combining it with the machine learning approach [77].

Techniques that depend on the detection of sickle cells’ mechanical deformability can only be used as a monitoring test in SCA, as they cannot indicate the disease’s severity in heterozygous states.

### 5.4. Lateral Flow Immunoassay

**Figure 2:**
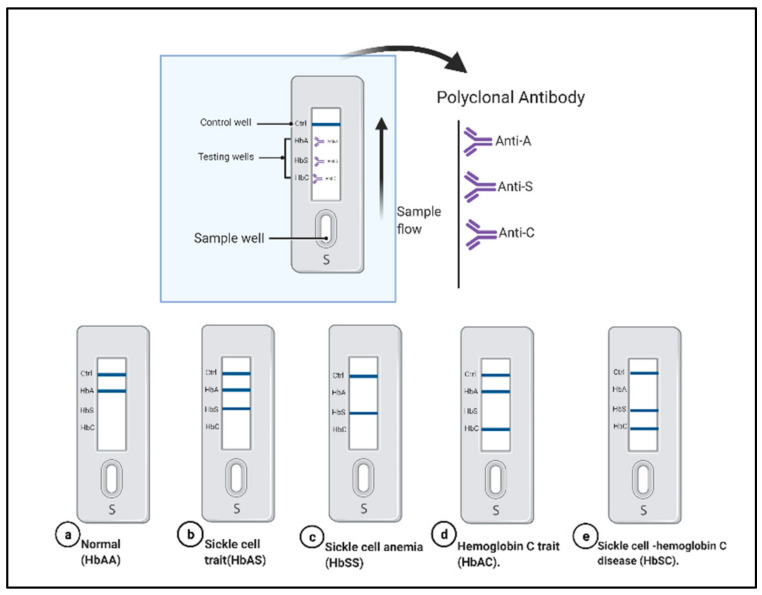
The Sickle SCAN based on lateral flow immunoassay to detect sickle-cell disease (SCD): normal hemoglobin HbAA (**a**); sickle cell trait HbAS (**b**); sickle cell anemia HbSS (**c**); hemoglobin C trait (**d**); and sickle cell-hemoglobin C disease HbSC (**e**). Created with BioRender.com.

Lateral flow assays (LFAs) are widely used as portable platforms in biomedical detection. Kanter et al. demonstrated a sensing platform called Sickle SCAN. It is used to detect normal hemoglobin HbAA, sickle cell trait HbAS, hemoglobin C trait HbAC, sickle-hemoglobin C disease HbSC, and hemoglobin C disease HbCC, as shown in Figure 2. The test used polyclonal antibodies on lateral flow chromaographic immunoassay against hemoglobin S, hemoglobin C, and hemoglobin A to detect the different types of SCD qualitatively. The polyclonal antibodies are attached on the test strip; the sample migrates in the absorbent pads, where the antibody conjugated to the nanoparticles binds to the hemoglobin; and then both migrate to the test strip. The hemoglobin binds with the corresponding antibody and produces blue lines, as shown in Figure 2 [78]. The Sickle SCAN cartridge contains four detection bands: the control band, normal HbA, HbS band, and HbC band. The test can be performed within minutes and costs a few dollars per test [78]. Mcgann et al. tested the validation of the Sickle SCAN assay to detect different hemoglobin. The test was performed using 139 whole blood samples (venous samples, dried blood spots, and spiked blood samples), and the results were compared to capillary electrophoresis (CZE) [79] The test’s accumulative sensitivity and specificity for HbSS were 98.4% and 98.6%, respectively. The cumulative sensitivity and specificity for the diagnosis of HbSC disease were 100%. A neonate sample with a high amount of HbF was tested and demonstrated that the detection of HbS or HbC was not affected by the high concentration of HbF. Furthermore, they examined the test’s storage condition and documented that the device can be stored at 37 °C for 30 days. However, the test suffers from some limitations such as misinterpretation of the result due to visual reading, cross-reactivity of the polyclonal antibody, and false-positive results in detecting the HbA heterozygous with HbS. The authors recommended another validation for the sickle SCAN in primary healthcare centers [79].

**Figure 3:**
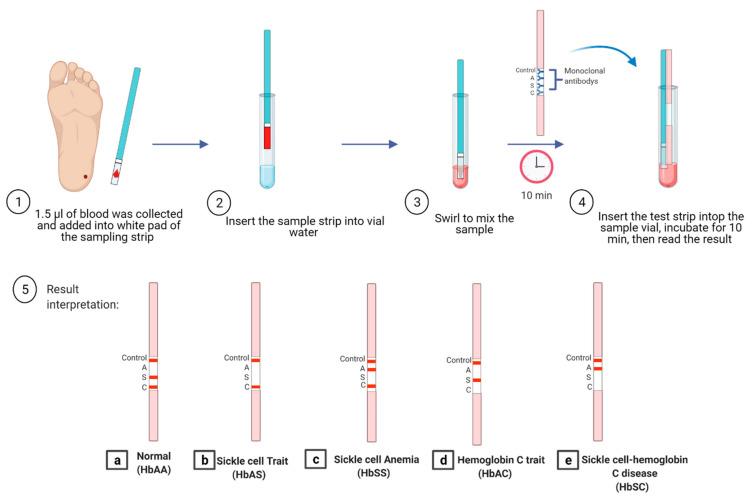
HemoTypeSC based on lateral flow immunoassay to detect SCD: normal hemoglobin HbAA (**a**); sickle cell trait HbAS (**b**); sickle cell anemia HbSS (**c**); hemoglobin C trait HbAC (**d**); and hemoglobin SC disease HbSC (**e**). Created with BioRender.com.

Another new device, called HemoTypeSC assay, has been developed based on competitive lateral flow immunochromatographic assay (LFA), which uses specific antibodies against hemoglobin A, S, and C (Figure 3). The test strip consists of laminated fiberglass, sample pads, nitrocellulose impregnated with the antibody for different Hb, and a cellulose wicking pad. The dried blood sample is diluted in detergent and non-specific blocking buffer. The test strip is placed in the test vial to migrate across the strip. As a result of this, a red line appears, as shown in Figure 3. It was reported that the result could be obtained in 20 min with 100% sensitivity [80]. The advantage of HemoTypeSC is that it can differentiate the normal Hemoglobin HbAA (three bands appear control S and C; the A band is absent) (Figure 3a), sickle cell trait HbAS (two bands appear; control, and C; the A, and S bands are absent) (Figure 3b), sickle cell anemia HbSS (three bands appear: control, A and C; the S band is absent) (Figure 3c) hemoglobin C trait HbAC (two bands will appear; control, and S; the C and A band are absent) (Figure 3d), and hemoglobin SC disease HbSC (two bands will appear; control and A; the S and C bands are absent) (Figure 3e). The advantages of the HemoTypeSC assay are that it is rapid, portable, cost-effective, and has promising characteristics to improve the diagnosis of SCD. However, it cannot detect HbF and HbA2 [80,81]. Nnodu et al. performed a validation study for HemoTypeSC by screening 1121 samples from babies, and they compared the results with HPLC. The discrepancies were confirmed by molecular diagnosis. The test showed 93.4% sensitivity and 99.9% specificity. The accuracy of HemoTypeSC was 99.1% [81]. Another validation study was performed in Uganda, where 1000 sickle cell disease phenotypes were tested and validated with CZE. The study reported ≥99.5% sensitivity and ≥99.9% specificity. Two samples with HbSS phenotype were missed and identified as HbAS. It was found that both patients had blood transfusions recently [82]. HemoTypeSC was tested in Côte D’Ivoire by analyzing 336 children: 100 children already diagnosed with hemoglobin disease and 236 as participants. The sensitivity and specificity of detection hemoglobin A, S, and C were 98.2% and 99.7%, respectively [83]. Unfortunately, the test was not able to differentiate between HbSS and sickle-β0-thalassemia, as well as showed misinterpretation of the result in patients who received blood transfusions recently [81].

### 5.5. Density-Based Separation

**Figure 4:**
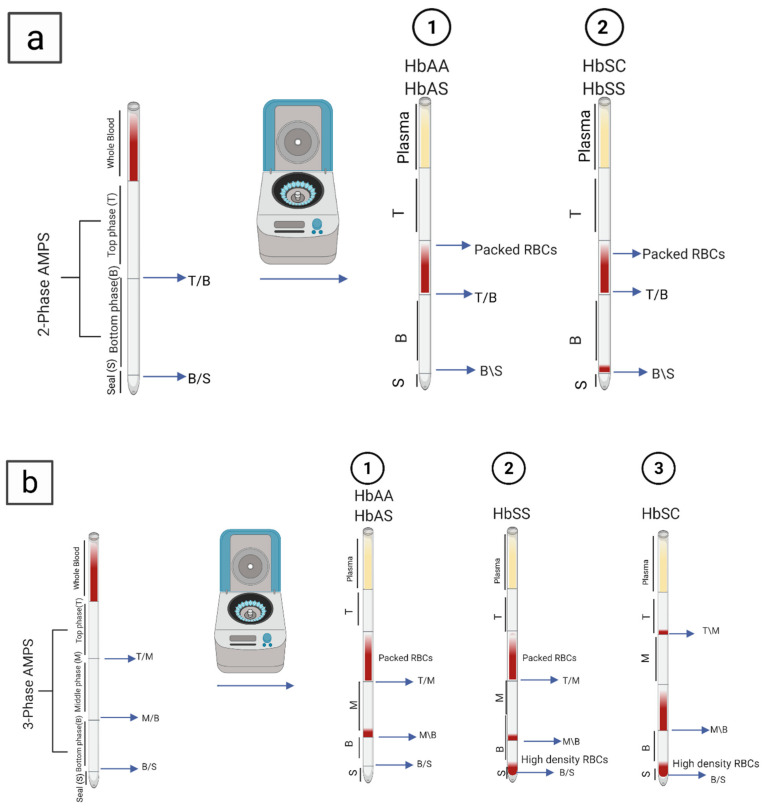
Multi-phase systems by cell density measurements (AMPS) to detects sickled RBCs: (**a**) two-phase AMPS HbAA, with HbAS (1) and HbSS and HbSC (2); and (**b**) three-phase AMPS, with HbAA and HbAS (1), HbSS (2), and HbSC (3). Created with BioRender.com.

Density-based separation detects sickled RBCs using aqueous multi-phase systems based on cell density measurements (AMPS). The dense sickle cells can be distinguished from normal cells by the two-phase AMPS system (Figure 4a, HbAA and HbAS (1), HbSS and HbSC (2)) with 90% sensitivity and 97% specificity. In contrast, the three-phase systems (Figure 4b) reported 91% sensitivity and 88% specificity [84]. The test only requires around 5 µL of the blood sample, which is mixed in capillary tubes with aqueous polymeric solutions. Upon 10 min centrifugation, the dense RBC precipitates and forms a layer in the bottom of the tube, which indicates the SCD [84]. The three-phase systems in Figure 4b were integrated with an optical reader to enable the distinction between HbSS and HbSC (Figure 4b, HbAA and HbAS (1), HbSS (2), HbSC (3)). The test is rapid and straightforward; however, this technique cannot distinguish between HbAA and HbAS (Figure 4b(1)). In addition, the accuracy of the test was compromised in cases with elevated HbF levels, such as in newborns and the Arab-Indian haplotype, due to the absence of dense RBCs. Furthermore, many health conditions, treatments, and medications can affect the RBC density and the test’s validity [18]. Kumar et al. validated the AMPS test, where they tested 505 samples. They reported 86% sensitivity and 60% specificity for SCD-AMPS-2. However, the sensitivity was dropped in children from 6 months to 1 year of age to 84%. Furthermore, the study documented 75% sensitivity and 60% specificity for SCD-AMPS-3, while the diagnostic accuracy was 69% for SCD-AMPS-3 and 77% for SCD-AMPS-2 [85].

A Portable smartphone platform has been developed to detect sickle cell using less than 1 μL of blood sample, based on the high density of sickle cells under hypoxic conditions. The blood sample is mixed with sodium metabisulfite to induce dehydration and cell sickling. The sample is illuminated with light-emitting diode (LED). Then, the image is magnified by an optical lens. Permanent magnets have been used for the magnetic levitation of the RBCs to observe the sickle cells image. The advantage of employing magnetic levitation is that it eliminates the need for the microscope or centrifugation [86].

### 5.6. Paper-Based Hemoglobin Solubility Test

**Figure 5:**
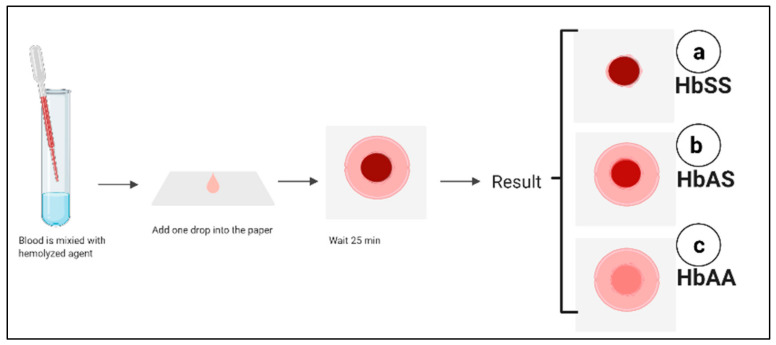
Paper-based hemoglobin solubility test. Created with BioRender.com.

It is a technique that depends on the filtration properties of the paper substrate and on the insolubility of the HbS, where it can be visually interpreted to detect the presence of hemoglobin S. In this test, one drop of the blood sample is mixed with a hemoglobin solubility agent with a ratio of 1:10. Then, the mixture is placed onto chromatography paper followed by staining. A different stained blood pattern forms based on the hemoglobin, as shown in Figure 5. These stains are used to determine HbSS (Figure 5a), the carrier HbAS (Figure 5b), and normal hemoglobin HbAA (Figure 5c). The test shows 94.2% sensitivity and 97.7% specificity for the visual detection of the HbS. Furthermore, the test can be performed within 20 min [87]. Moreover, the test’s sensitivity is increased when combined with an image analysis algorithm, which quantifies the Hb in the sample based on the intensity of the color in the center spot. This test has several advantages: simple fabrication, easy to use as it needs single step, affordable, and low-cost (costs less than a dollar per test) [87,88]. However, this test is affected by the blood clotting, preventing the blood from wicking through the paper substrate. Furthermore, the test is unable to differentiate between HbSC and HbAS. Finally, this test is not reliable for newborns due to a high level of HbF, which prevents the polymerization and precipitation of hemoglobin S [87,88]. Validation of the paper-based screening test was performed by Piety et al., who tested 226 samples and compared the results with IEF electrophoresis to distinguish HbAS and HbSS from HbAA patients. The study reported 94.2% sensitivity, 97.7% specificity, and 96.9% accuracy [89]. The authors employed automated image analysis C-index to distinguish between HbSC and HbAS. They demonstrated 100% sensitivity and 59% specificity. The high sensitivity but relatively low specificity indicated that the C-index could be used to exclude patients without the disease. Furthermore, this test cannot detect sickle cell disease in neonates due to the high level of HbF [89].

A microchip electrophoresis paper-based has been developed to identify and quantify hemoglobin types, including HbA, A2, F, S, and C [90]. This microchip is named HemeChip platform, and it consists of a cartridge where the hemoglobin variants are isolated, pictured, and tracked during electrophoresis. HemeChip is based on separating the hemoglobin using cellulose acetate electrophoresis [18,90]. The HemeChip test is performed by mixing the blood sample with deionized water, and then the microchip is placed inside the HemeChip reader and an electrical field is applied, which results in separating the Hb. As a result, each Hb moves a unique distance across the paper strip. Real-time images are taken during the test and analyzed automatically using custom built-in software [90]. This test has a number of advantages such as low-cost, disposable, portable, high accuracy, easy to perform, and can be completed in less than 10 min [18].

### 5.7. Sensors Based Techniques

#### 5.7.1. Fluorescence Based Optofluidic Resonator

An optofluidic resonator based on the waveguide has been used to identify the interaction of Fe^2+^ and Fe^3+^ within protoporphyrin IX in phosphate buffer saline. The metal clad waveguide optofluidic resonator is used for the real-time detection using a small sample volume. The Fe^2+^ molecule shows a fluorescent peak which corresponds to the normal hemoglobin, while the Fe^3+^ fluorescent peaks are correlated with the S hemoglobin of sickle-cell disease patients [91].

#### 5.7.2. Sensors Based on Electrical Impedance Signal 

The electrical impedance signal is a sensitive indicator of the disease severity and red blood cell sickling events. Combining the microfluidic chip with the electrical impedance provides a promising method for identifying sickling and unsickling processes in SCD patients [92]. Liu et al. developed a microfluidics-based electrochemical impedance sensor to determine the SCD red blood cells’ electrical properties under oxygen control conditions. The electrical impedance of sickle cells shows a significant difference between normoxic and hypoxic conditions [93]. These methods have been developed to monitor the cell sickling, and they do not accurately describe the severity of heterozygosis SCD.

#### 5.7.3. Quartz Crystal Microbalance (QCM)

The quartz crystal microbalance (QCM) sensor mainly depends on the change in the frequency of a quartz crystal resonator due to the mass variation at the crystal surface [94]. Quartz crystal microbalance has been used to determine the morphological change of RBCs. Combining quartz crystal microbalance and a novel mathematical model provides complete information about the changes in the RBC’s elasticity. This sensor can differentiate between normal biconcave discoid RBCs and sickled cells and can be used as monitoring test [95].

#### 5.7.4. Genosensors

Brazaca et al. demonstrated an electrochemical nanosensor to detect SCA trait based on the immobilization of mutated single-strand DNA on a gold electrode. The hybridization between the probe and target DNA is measured using electrochemical impedance spectroscopy (EIS). The sensor allows the detection of the SCA trait individuals and provides genetic consulting [96]. An oligonucleotide sensor has been developed for the detection of β-globin point mutation caused by sickle cell anemia. The sensor employs luminescence resonance energy transfer between photon upconverting nanoparticles (donor) and conventional fluorophore (acceptor). The sensor can determine the matched target in random oligonucleotides sequences with high sensitivity and specificity [97]. SPR biosensor has been documented to detect point mutations in the HBB gene. The technology depends on the immobilization of wild-type and βS mutated probes. The probes can differentiate between wild-type alleles and mutated ones. This technique is affordable and allows real-time detection of single-point mutations, responsible for sickle cell disease, making it suitable for effective prenatal diagnosis. This sensor needs a PCR product, which limits the ability to use it at POC [98]. The specificity of the genosensors depends on the probe design and the immobilization methods. These sensors can be improved to detect different types of SCD accurately and not be affected by the presence of different hemoglobin fractions.

### 5.8. The Pyrosequencing Technique

The pyrosequencing technique (PyS) has been used to identify different types of homozygous or heterozygous SCD. This test aims to sequence hemoglobin of a small number of patients accurately to differentiate between βSβS and Sβ0 thalassemia and detect HbC mutations. The difference in the mutations is only in a single nucleotide, which is hard to detect with conventional tests. PyS was compared with sanger sequencing, and it was found to be able to correctly identify βSβS with 98.7% accuracy, sickle cell-hemoglobin C disease with 98.7%, and the heterozygous with 92.2%. It was also proven to be satisfactory for β+ and β0 mutations found in SCD patients. However, several samples were wrongly classified by PyS, due to the low level of the pyrogram peaks [99]. This sequencing technique has significant potential to provide an accurate diagnosis of SCD disease and differentiate between the types of the disease, and it can be used in neonatal screening.

## 6. Conclusions

Sickle cell disease diagnosis has been part of the clinical laboratory for almost a century. In the last few decades, studies have enhanced the knowledge about the physiology and biochemistry of these genetic disorders to improve the detection and help in developing advanced strategies to combine molecular techniques and classical biochemical methods. Regardless of the methods implemented, the outcomes must be correlated with the clinical picture. To conclude, most hemoglobin variants can be identified and controlled by RBC indices, HPLC results, and family studies. However, the drawbacks associated with the diagnostic approaches should be known to prevent a false-negative diagnosis. Genetic tests are recommended to validate borderline cases and detect unusual and novel variants. Recently, different portable and rapid devices have been described to diagnose SCD, including platforms based on immune assay, density-based separation, and sensor-based technologies. 

**Table 1:** Technologies for sickle-cell disease (SCD) diagnosis and monitoring.

Technique	Sensitivity	Specificity	Accuracy	Advantages	Disadvantage	Result	Ref.
Peripheral blood smear (PBF)	35.0%.	96.7%	90.5%	Simple preparation, inexpensive, Turnaround time (TAT) is 44 min	Dependence on the pathologist’s skills, does not differentiate between different types of SCD	Detect sickle cells	[22]
Solubility and Sickling	Sickling: 65.0% Solubility: 45.0%.	Sickling: 95.6% Solubility: 90.0%.	Sickling: 92.5% Solubility: 85.5%.	Easy, inexpensive, fast, affordable, TAT 38 min for sickling, TAT for solubility 70 min	Testing newborns shows false-negative result, does not differentiate between SCD types	Detect the sickling event.	[27]
Capillary electrophoresis	Not reported	Not reported	Not reported	Reliable, ability to distinguish most types of sickle cell disease including heterozygous.	Expensive, requires skilled technicians	identify and quantify HbF, Hb A, Hb A2, Hb S, Hb C, Hb Barts and other	[36]
Isoelectric focusing (IEF)	Not reported	Not reported	Not reported	Detect HbS and HbA easily in a high concentration of HbF, Hb D-Punjab easily separated from HbS, need small volume of the sample, able to use dried blood spot, TAT is 45 min.	Expensive, requires highly trained staff to interpret the results.	Hb A, Hb F, Hb C, Hb S, Hb E and Hb O Arab	[38]
High-performance liquid chromatography (HPLC)	Not reported	Not reported	Not reported	Reliable, ability to distinguish most types of sickle cell disease including heterozygous, fully automated	Misdiagnoses the new variants that mimic HbS, Expensive and needs trained personnel, not practical in limited resources areas	Detect Hb F, Hb A2, Hb S, Hb C, Hb Barts, and other Hb variants.	[18,44]
Amplification-refractory mutation system (ARMS) polymerase chain reaction (PCR) for prenatal analysis	75%	Not reported	Not reported	Simple, can be used for prenatal diagnosis	Low sensitivity, maternal cell DNA contamination	βSβS βAβS βAβA	[49]
Allele-Specific Recombinase Polymerase Amplification	100%	βA: 94.7% βS:97.1%	<95%	Affordable, rapid (less than 30 min), low-cost, accurate	This test is difficult to design, missing some single nucleotide polymorphisms (SNPs), costly and laborious assay	βA βS	[100] [101]
**Emerging technologies**							
Image processing technique	96.55%	Not reported	95%	Automated method to detect sickle cells, minimize the error of dependence on the naked eye	Cannot distinguish between different types of SCD, cannot be used to determine the severity of the disease, affected by different conditions that can affect the red blood cells (RBCs) number as in blood transfusion, expensive, needs special equipment such as camera connected to microscope	Detect Sickling RBCs	[66]
Propose deep learning models	Not reported	Not reported	99.54%	Indicate the sickle RBCs automatically in one shot, minimize the error of dependence on the naked eye	Cannot distinguish between different types of SCD, cannot be used to determine the severity of the disease, affected by different conditions that can affect the RBCs number as in blood transfusion, needs special equipment such as camera connected to microscope., time consuming, ignore other cells which leads to false diagnosis	Detect Sickling RBCs	[67]
Smartphone microchip with microscope and machine learning algorithms	Not reported	Not reported	Not reported	Can be used as point of care (POC) to monitor the diseases severity, reduce the cost	Test is based on the morphology of the RBCs, cannot distinguish between different types of SCD, affected by different conditions that can affects the RBCs morphology	Detect Sickling RBCs	[68]
Electrical impedance microflow cytometry	91%	86%	Not reported	Used to monitor the sickling events accurately	Does not differentiate between different type of SCD, need to be validated	Electrical impedance of the sickle cells Detect Sickling RBCs	[19] [73]
Imaging flow cytometry	Not reported	Not reported	Not reported	Robust test, can be automated to correlate the percentage of HbF and the percentage of sickled cells, biomarker of disease severity	Effected by agents that reduce polymerization of HbS, laborious	Used to quantify sickled cells	[71]
Optical tweezer to capture red blood	Not reported	Not reported	Not reported	Can be a monitor test, simple	Cannot indicate the severity of the disease in heterozygous states	Measuring red blood cell elasticity	[102]
Photoacoustic Flow cytometry	Not reported	Not reported	Not reported	Simple, low-cost, uses cellphone-like camera.	It is not clear if it can be used to monitor the disease severity, cannot distinguish between sickle cells trait and sickle cell disease	Determine the RBCs Sickling	[72]
lateral flow Immunoassay sickle SCAN	90%	100%	98%	Simple, rapid	Relies on polyclonal antibody, more expensive, low specificity and cross reactivity, qualitative test, the intensity of band shows inconsistency, does not identify hemoglobin F, limit of detection of Hb A is 2%	Identify HbC and HbS	[103]
lateral flow Immunoassay HemoTypeSC	93.4%	99.9%	99.1%	Cost-effective, rapid, POC	Cannot detect all hemoglobin variants, does not differentiate between HbSS and sick-le-β0-thalassemia, misinterpretation of the result in cases with recent blood transfusion	HbAA, HbAS, HbAC, HbSC, and HbCC	[80,81]
HemeChipMicro-elecrophoresis assay	100%	HbSS 98.7% Other type 100%	100%	Reliable. POC, inexpensive, simple	Interpretation requires skills, the need for web-based image for automated results	SCD-SS, SCD-SC, and SCD Trait Hb E Disease	[90]
SCD-AMPS 2-phase	90%	97%	77%	Inexpensive, simple POC	Interpretation is difficult, less reliable, affected by different conditions that decrease the number of dense cells, may not be appropriate for neonatal screening, low sensitivity and specificity	Identifies Hb S and Hb A	[18,84,85]
SCD-AMPS 3-phase	91%	88%	69%			Identifies Hb S, Hb A and Hb C	[18,84,85]
Paper-based hemoglobin solubility test	94.2%	97.7%	96.9%	Simple, rapid, inexpensive POC, does not need trained personal	Difficult to distinguish HbAS (trait) from HbSC, humidity can affect the test result, low sensitivity and specificity	Diagnosis of HbSS	[89]
Quartz crystal microbalance (QCM) sensor	Not reported	Not reported	Not reported	Reliable, simple, POC, low-cost	Not a diagnostic test	Determine RBC’s elasticity	[95]
Electrochemical genosensor	1.23 × 105 ohmLmmol-1 cm-2	Not reported	Not reported	Simple, low cost, POC	Determination of SCA trait only	Detect βAβS	[96]
Surface plasmon resonance-based biosensor	Not reported	Not reported	Not reported	Simple, rapid	Needs PCR product, needs to be validated	Detect βSβS	[98]
The Pyrosequencing technique (PyS)	98.2%	Not reported	Sickle cell anemia 98.7% sickle cell- hemoglobin C disease with 98.7%, and the heterozygous with 92.2%,	Diagnose heterozygous SCD, simple, fast, low cost, suitable for large scale	Misclassification, false negativity, depends on primer design	Detect βSβS, Sβ0 thalassemia, Sβ+ thalassemia, and aickle-hemoglobin C	[99]
